# Bicuspid aortic valve annulus: assessment of geometry and size changes during the cardiac cycle as measured with a standardized method to define the annular plane

**DOI:** 10.1007/s00330-021-07916-8

**Published:** 2021-04-24

**Authors:** Sara Boccalini, Lidia R. Bons, Allard T. van den Hoven, Annemien E. van den Bosch, Gabriel P. Krestin, Jolien Roos-Hesselink, Ricardo P. J. Budde

**Affiliations:** 1 Department of Radiology, Erasmus Medical Center, P.O. Box 2040, 3000 CA Rotterdam, The Netherlands; 2 Department of Cardiology, Erasmus Medical Center, P.O. Box 2040, 3000 CA Rotterdam, The Netherlands

**Keywords:** Bicuspid aortic valve, Cross-sectional anatomy, Cardiovascular physiological phenomena, Computed tomography angiography, Transcatheter aortic valve replacement

## Abstract

**Purpose:**

Bicuspid aortic valve (BAV) is a complex malformation affecting not merely the aortic valve. However, little is known regarding the dynamic physiology of the aortic annulus in these patients and whether it is similar to tricuspid aortic valves (TAV). Determining the BAV annular plane is more challenging than for TAV. Our aim was to present a standardized methodology to determine BAV annulus and investigate its changes in shape and dimensions during the cardiac cycle.

**Methods:**

BAV patients were prospectively included and underwent an ECG-gated cardiac CTA. The annulus plane was manually identified on reconstructions at 5% intervals of the cardiac cycle with a new standardized method for different BAV types. Based on semi-automatically defined contours, maximum and minimum diameter, area, area-derived diameter, perimeter, asymmetry ratio (AR), and relative area were calculated. Differences of dynamic annular parameters were assessed also per BAV type.

**Results:**

Of the 55 patients included (38.4 ± 13.3 years; 58% males), 38 had BAV Sievers type 1, 10 type 0, and 7 type 2. The minimum diameter, perimeter, area, and area-derived diameter were significantly higher in systole than in diastole with a relative change of 13.7%, 4.8%, 13.7%, and 7.2% respectively (all *p* < 0.001). The AR was ≥ 1.1 in all phases, indicating an elliptic shape, with more pronounced flattening in diastole (*p* < 0.001). Different BAV types showed comparable dynamic changes.

**Conclusions:**

BAV annulus undergo significant changes in shape during the cardiac cycle with a wider area in systole and a more elliptic conformation in diastole regardless of valve type.

**Key Points:**

*• A refined method for the identification of the annulus plane on CT scans of patients with bicuspid aortic valves, tailored for the specific anatomy of each valve type, is proposed.*

*• The annulus of patients with bicuspid aortic valves undergoes significant changes during the cardiac cycle with a wider area and more circular shape in systole regardless of valve type.*

*• As compared to previously published data, the bicuspid aortic valve annulus has physiological dynamics similar to that encountered in tricuspid valves but with overall larger dimensions.*

**Supplementary Information:**

The online version contains supplementary material available at 10.1007/s00330-021-07916-8.

## Introduction

With an estimated incidence of 0.4–2% in the general population, bicuspid aortic valve (BAV) is likely the most common congenital cardiac anomaly [1–4]. BAV are prone to precocious stenosis and regurgitation, often requiring an intervention early in life [5]. Nevertheless, this disease does not affect exclusively the valve but the entire valvular apparatus and aorta resulting in a complex disease. In addition, these structures are exposed to continuous and intense forces during the cardiac cycle resulting in arduous analysis and classification.

For instance, the annulus has been shown to have an elliptical shape that undergoes conformational changes during the cardiac cycle in a normal as well as stenotic tricuspid aortic valve (TAV) using ECG-gated CT imaging [6, 7]. Furthermore, most sizing parameters for the TAV aortic annulus show significant changes between diastole and systole [8]. If, and to what extent, such dynamic geometry changes also occur in the BAV annulus is unknown.

The broad variability in cusp morphology of BAV results in the cumbersome and, more importantly, sometimes impossible identification of the annulus plane as defined based on the standard anatomy of TAV. Therefore, a specific and standardized method for the definition of the annulus plane in BAV patients is strongly needed but lacking.

Comprehension of the physiology is likely to lead to adapted and specific pre-procedural evaluation and improved therapeutic strategies of BAV patients. The distortion of the valve apparatus in BAV patients has led to the exclusion of patients with this anomaly from earliest studies assessing safety and results of transcatheter aortic valve implantation (TAVI), due to concerns about deformation and malposition of the implanted valve [9–11]. Yet, in the last few years, the procedure has been performed with an increasing frequency in BAV patients. In fact, although with limitations due to the inclusion of small populations, later investigations have shown that TAVI is in fact feasible also in the BAV population and, furthermore, has results comparable to those obtained in cohorts with TAV [12–15].

Based on a revised method to determine the annulus plane, we assessed the presence and magnitude of geometrical and dimensional changes of the annulus during the cardiac cycle using ECG-gated CT as well as investigated if different BAV subtypes present different patterns.

## Materials and methods

### Study population and BAV classification

Sixty consecutive patients with a BAV were prospectively included as part of a clinical study at the Erasmus Medical Center, Rotterdam, The Netherlands, between November 2014 and March 2016 as previously reported [16, 17]. The local medical ethics committee approved the study and all patients provided written informed consent. As part of the study, an ECG-gated contrast-enhanced CT of the heart was performed.

The morphology of the aortic valve was classified as previously described by Sievers et al [1] based on images obtained with echo, CT, and magnetic resonance.

### CT scans

Contrast-enhanced CT scans were acquired with a third-generation dual-source CT scanner (Somatom Force, Siemens Healthineers) with the parameters summarized in Supplemental Material Table I. All scans were performed with retrospective ECG gating, 192 × 0.6mm collimation and 250 ms rotation time. Reconstructions of 1.5 mm thickness were performed throughout the entire cardiac cycle at 5% intervals resulting in 20 image datasets per patient.

### Annulus analysis

A radiologist with > 5 years of experience in cardiovascular radiology (S.B.) defined the annulus plane and semi-automatically measured the annulus dimensions on the CT scans for each of the 20 reconstructed phases.

The beginning of the systole was identified as the first phase where the aortic valve was opening/was open and its end as the first phase where the valve was closed.

### Annulus plane definition

**Fig. 1:**
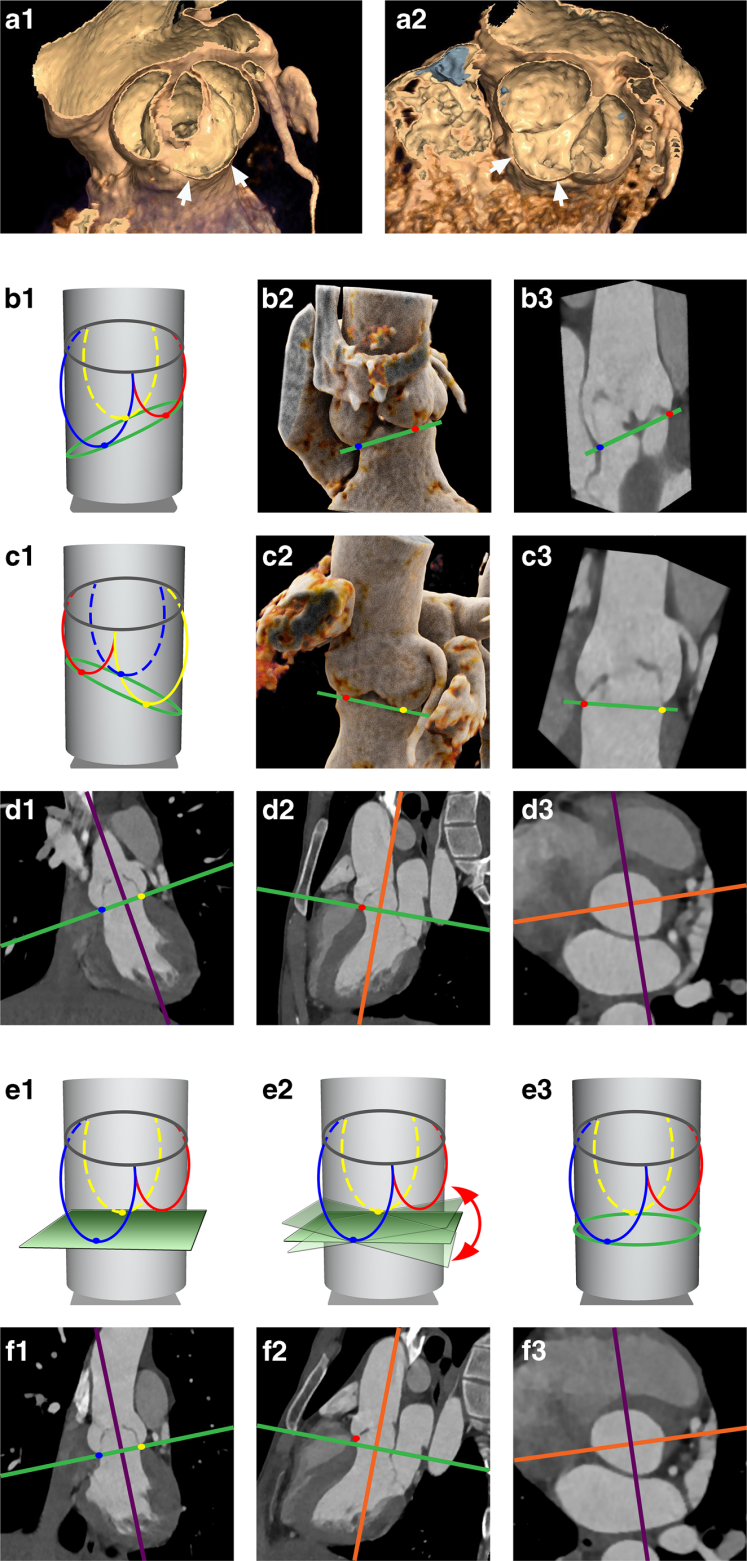
Determination of the annulus in patients with BAV type 1 with asymmetric cusps. (**a)** Volume rendered (VR) reconstructions in systole (**a1**) and diastole (**a2**) demonstrating the asymmetry of the sinuses due to the smaller dimensions of the right coronary cusp (RCC) (arrows). (**b**–**d**) Wrong annulus plane definition based on the three hinge points. In (**b1**) and (**c1**) schematic representations of the LVOT, sinuses of Valsalva (red, yellow, blue lines) and sinotubular junction (gray line) where the annulus plane (green line) was identified as the plane passing through the three hinge points (hinge point of the RCC in red; hinge point of the non-coronary cusp (NCC) in blue; hinge point of the left coronary cusp (LCC) in yellow). In (**b2**–**b3**) and (**c2**–**c3**), VR reconstructions showing the angulation between the centerline passing through the LVOT/aortic root and the line (**b2**–**b3** and **c2**–**c3**; green lines) passing through the hinge points of the LCC and the NCC (**b**) and RCC (**c**), respectively. In (**d1**) and (**d2**), MPR showing the position of the axis in the longitudinal planes when identifying the plane passing through the three hinge points (**d3**). (**e**–**f**) Correct annulus plane definition. At first, one plane passing through the two hinge points of the two biggest cusps is identified (**e1**); then, this plane is tilted along the only still undetermined direction (**e2**) until the minimum cross-sectional area and/or a plane perpendicular to the centerline is obtained (**e3**). In (**f1**) and (**f2**), MPR showing the position of the axis in the longitudinal planes when determining the plane passing through the two hinge points and then make adjustments by tilting the violet and orange axis to identify the smallest possible area and a plane perpendicular to the centerline (**e3**)

**Fig. 2:**
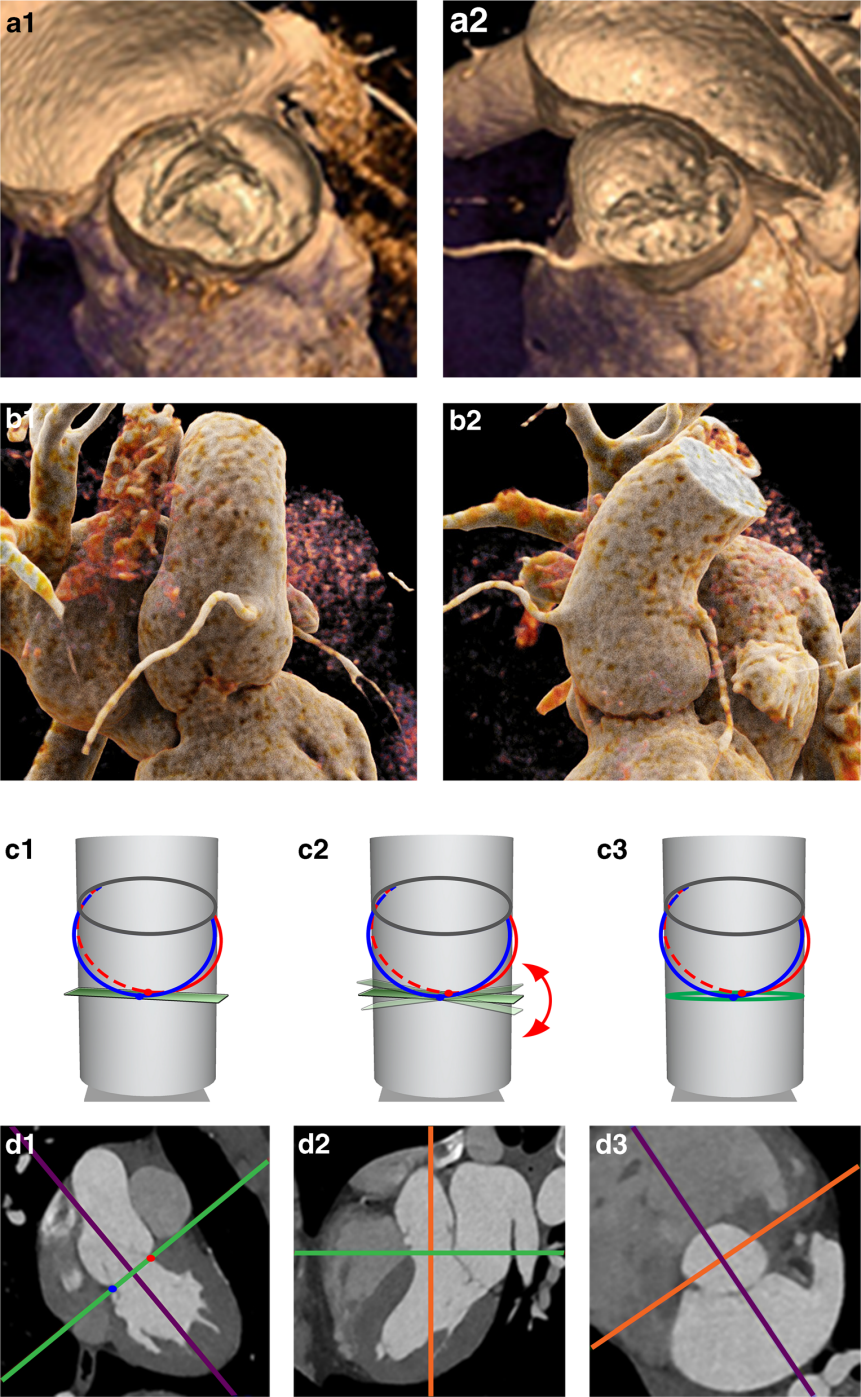
Determination of the annulus plane in patients with BAV type 0. (**a**) VR reconstructions in systole (**a1**) and diastole (**a2**) showing the presence of only two cusps. (**b**) 3D anatomy of the aortic root. (**c**–**d**) One plane passing through the two hinge points of the two cusps is defined (**c1**); then, this plane is tilted along the only still undetermined direction (**c2**) until the minimum cross-sectional area and/or a plane perpendicular to the centerline is obtained (**c3**). In (**d1**) and (**d2**), MPR showing the position of the axis in the longitudinal planes when determining the annulus plane (**d3**) as detailed above

Images were exported to a commercially available workstation (IntelliSpace Portal, Philips) to allow for manual multiplanar reconstructions (MPR). The annulus plane was identified with a different methodology depending on the morphology of the bicuspid valve. In BAV with three equally/similarly sized cusps (Sievers type 1 and 2), the annulus plane was identified as the plane passing through the three hinge points (defined as the lowest insertion point of the valve leaflet on the aortic wall) as indicated by guidelines for TAV [18]. In case the fused cusps had different dimensions (Sievers type 1) or there were only two cusps (Sievers type 0), the annulus plane was identified with a newly developed approach consisting of multiple steps based on a combination of previously published definitions of the annulus: first, one of the planes passing through the hinge point of the non-fused cusp and the hinge point of the bigger one of the two fused cusps (or through the two hinge points in valves type 0) was identified; then, this plane was tilted until the minimum annulus area at that level was identified [19–22]; if this plane was considered to be too steeply angled in respect to the LVOT and the sinuses, a compromise between the plane with the smallest diameter and a plane perpendicular to the centerline at this level was reached [23, 24] (Figs. 1 and 2). The plane was checked and adapted when necessary for each of the analyzed phases to account for displacement of the annulus due to the contraction of the heart.

### Annulus measurements

**Fig. 3:**
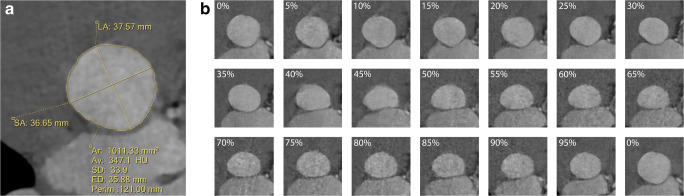
Annulus measurements. (**a**) Measurement of the annulus parameters (maximum and minimum diameters, perimeter, area, and area-derived diameter) based on the semi-automatically defined contour. (**b**) A case example showing all the 20 phases analyzed in a patient with BAV type 1 LR. The annulus shows a more circular shape in systole and a more elliptic shape during diastole

The annulus measurements were performed semi-automatically with the “edge finder” tool available in the reconstruction software (IntelliSpace Portal). With this tool, two seed points are placed somewhere inside the annulus and in the tissues surrounding the aortic root, respectively. Thereafter, the software automatically defines the contour of the annulus where there is a sharp transition of Hounsfield unit values between the contrast-enhanced lumen and surrounding tissues. For each phase, the automatically traced contour was checked and adjusted when deemed necessary. The contour was modified to encircle the inner edge of small calcifications and to extend over big calcifications in continuity with the adjacent outline. The maximum diameter, the diameter perpendicular to the maximum (referred to as “the minimum diameter”), the area, and the perimeter were recorded (Fig. 3). The average diameter was calculated as the average of the maximum and minimum. The effective diameter was derived from the area [18]. The asymmetry ratio (AR) and relative area (RA) were calculated as previously defined [6]. The AR was defined as the ratio between the maximum and the minimum diameter. The RA was calculated as the ratio between the area at each individual phase and the average area of all phases throughout the cycle.

### Image quality assessment

The image quality of each phase at the level of the annulus was subjectively assessed based on a 2-point scale (insufficient quality; acceptable to perfect quality) based on the following criteria: the definition of the cusps and hinge points and subsequent reliability in the identification of these landmarks; the presence of motion artifacts; the adequate opacification of the LVOT and aortic root. The phases with insufficient quality were excluded from further analysis.

### Intra- and inter-observer variability

The same observer re-measured all phases of one-third of the patients (*n* = 18) at least 1 month after the first measurement. A second observer with 2 years’ experience in cardiovascular imaging (L.B.) blinded to previous results analyzed 20 phases of 6 patients.

### Statistical analysis

Continuous data are presented as mean ± standard deviation (SD). Student’s *t*-test for paired samples and Wilcoxon signed-ranks tests were employed to assess differences during the cardiac cycle for all annular parameters. Data were examined with the Kolmogorov-Smirnov test to ensure normal distribution. A one-way ANOVA model with Bonferroni correction and Welch’s test was used to investigate differences between valve types. Random distribution of missing data was tested with Little’s test. A *p* value of < 0.05 was considered as statistically significant.

## Results

### Patients and valve characteristics

Of the 60 eligible patients, 5 were excluded because of overall insufficient image quality (*n* = 1), not all reconstruction phases available (*n* = 3), and the presence of a large septal aneurysm (*n* = 1).

In total, 55 patients were included (mean age: 38.4 ± 13.3 (21–66); males: 32 (58%)) of whom 10 had a Sievers type 0 BAV, 38 a type 1 BAV, and 7 a type 2 valve. Patients’ characteristics are summarized in Table 1.

**Table 1:** Patient characteristics

	Total (*n* = 55)	Sievers type 0 (*n* = 10)		Sievers type 1 (*n* = 38)		Sievers type 2 (*n* = 7)
		ap (*n* = 6)	lat (*n* = 4)	LR (*n* = 30)	RN (*n* = 8)	
Male*	32 (58%)	4 (67%)	3 (75%)	17 (57%)	4 (50%)	4 (57%)
Age in years*	38.4 ± 13.3 (21–66)	41.5 (30.5)	35 ± 7.8 (24–41)	37.5 (27)	36.7 ± 12.7 (22–54)	33.8 ± 8.5 (22–43)
Height in m*	1.7 ± 0.2 (1.3–2)	1.8 ± 0.1 (1.6–1.9)	1.8 ± 0.1 (1.6–1.9)	1.7 ± 0.2 (1.3–2)	1.7 ± 0.2 (1.4–2)	1.8 ± 0.2 (1.5–2)
Weight in kg*	75 ± 16 (48–132)	76.7 ± 15.9 (55–95)	77.7 ± 10 (69–92)	72 (19.5)	67.7 ± 16.1 (48–90)	77.7 ± 18.4 (52–106)
BMI (mean, SD, range)*	24.3 ± 3.2 (17.9–34)	24.1 ± 1.1 (22.3–25.8)	24.5 ± 2.6 (20.9–26.6)	24.9 ± 3.8 (17.9)	23.1 ± 2.5 (18.3–26.8)	23.2 ± 2.6 (20–27)
Blood pressure (the day of the CT scan)*						
- Max	124 ± 16 (94–170)	130 ± 22 (102–170)	138 ± 27 (107–161)	124 ± 15 (94–156)	119 ± 8 (109–130)	117 ± 14 (99–134)
- Min	81 ± 11 (64–117)	81 ± 10 (69–94)	94 ± 24 (66–117)	83 ± 9.8 (65–104)	76 ± 7 (67–91)	75 ± 8 (64–89)
Aortic valve dysfunction*						
- Insufficiency (at least mild)	9 (16%)	1 (17%)	0	3 (10%)	2 (25%)	3 (42%)
- Stenosis (peak velocity > 2.5 cm/s)	24 (44%)	2 (33%)	1 (25%)	8 (27%)	7 (87%)	6 (86%)
- Severe stenosis (peak velocity > 4cm/s)	6 (11%)	1 (17%)	0	1 (3%)	3 (4%)	1 (14%)
Additional valve related events						
- Previous endocarditis	1 (2%)	0	0	0	0	1 (14%)
- Previous balloon-dilation	5 (9%)	0	0	3 (1%)	2 (25%)	0
Congenital aortic valve or aortic diseases						
- Subvalvular stenosis	1 (2%)	0	0	1 (3%)	0	0
- Turner syndrome	9 (6%)	0	1 (25%)	6 (20%)	1 (12%)	1 (14%)
- Coarctation	5 (9%)	0	2 (50%)	3 (1%)	0	0
- Turner syndrome and coarctation	2 (4%)	1 (17%)	0	1 (3%)	0	0

Data are presented as: mean, SD, range; median and interquartile range; number, percentage. Percentages were rounded to the nearest integer. ap = BAV Sievers type 0 with the maximum diameter of the valve opening oriented along an anteroposterior axis. lat = BAV Sievers type 0 with the maximum diameter of the valve opening oriented along a latero-lateral axis. LR = BAV Sievers type 1 with fusion of the left coronary cusp (LCC) and the right coronary cusp (RCC). RN = BAV Sievers type 1 with fusion of the RCC and the non-coronary cusp (NCC). *No statistically significant differences in age, sex, height, weight, BMI, blood pressure, or aortic valve pathology were found between patients with valve types 0, 1, and 2

In 9 patients with Sievers type 1, the new method for the definition of the annulus plane based on two hinge points was used. Ten patients had calcifications of the annulus that were of considerable amount in only one case.

### Variation of annulus parameters during the cardiac cycle

Out of a total of 1100 phase datasets (55 patients with 20 reconstructed phases each), 71 (6.4%) were excluded due to insufficient quality. The excluded datasets were randomly distributed over the cardiac cycle (*p* = 0.6).

**Fig. 4:**
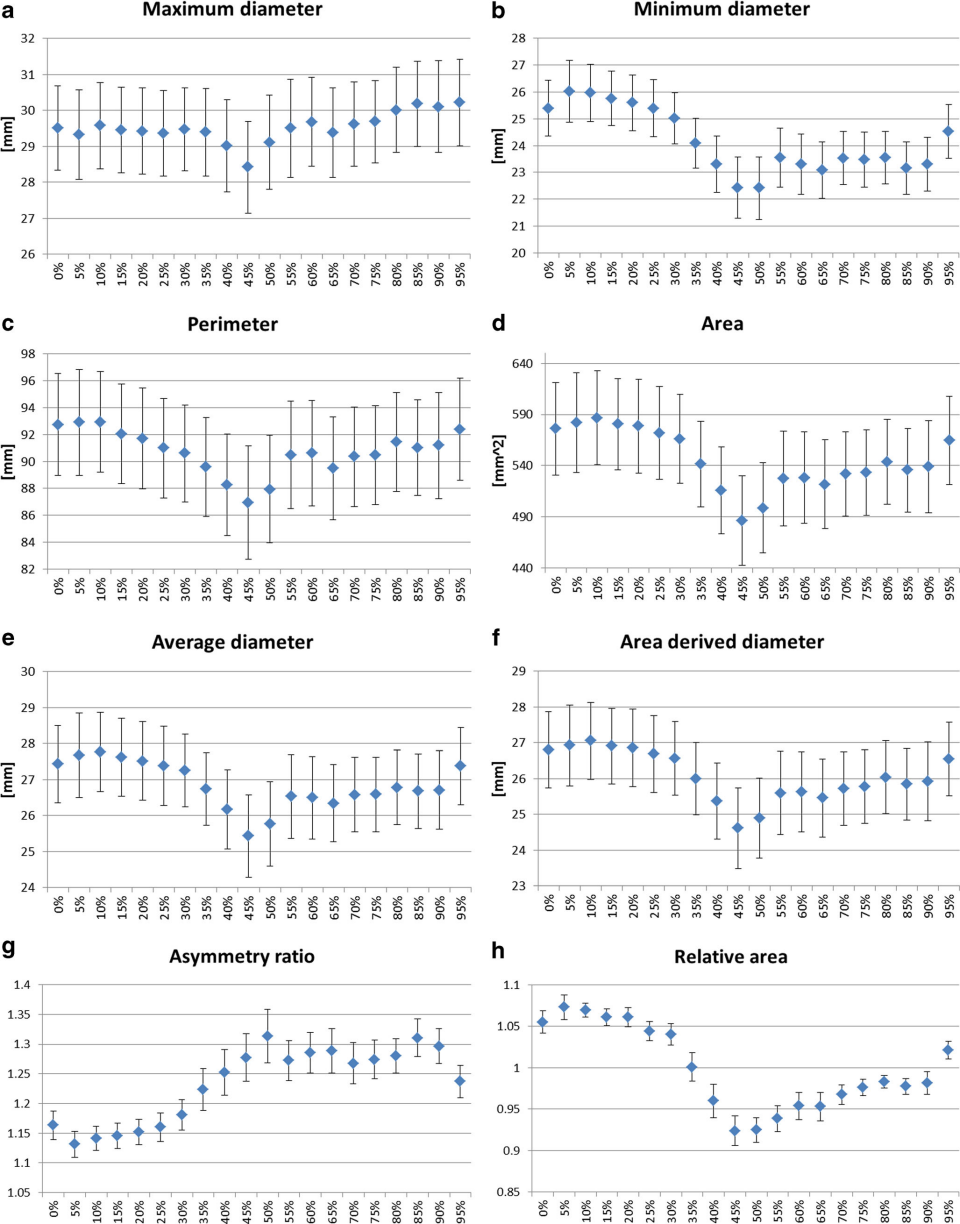
Mean annulus parameters per cardiac phase. Vertical bars indicate 95% confidence intervals (CI)

All of the measured annulus parameters presented statistically significant differences during the cardiac cycle (Table 2 and Fig. 4). The minimum diameter, the average diameter, the area, the perimeter, and the area-derived perimeter demonstrated the highest values during early systole (5–10% of the cardiac cycle) with a significant reduction in diastole of 13.7%, 6.7%, 13.7%, 4.8%, and 7.2%, respectively (all *p* values < 0.001). The RA was > 1 during systole (0–30%) and in late diastole (90–100%) (*p* < 0.001). The maximum diameter showed the highest value in diastole (*p* < 0.001). The AR was ≥ 1.1 in all phases indicating an elliptic shape of the annulus throughout the entire cardiac cycle. The annulus demonstrated significant deformation with increased elliptic morphology during diastole (relative change of 13.9%; *p* < 0.001).

**Table 2:** Changes of the annulus parameters values during the cardiac cycle

	Max value*	Min value*	Relative difference [%]†ǂ (± SD of the difference)	*p* valueǂ
				Min vs Max
Maximum diameter [mm]	30.2 ± 4.5	28.4 ± 4.2	3.6% (± 3.9%)	< 0.001
Minimum diameter [mm]	22.4 ± 4.1	26.0 ± 4.1	13.8% (± 8.4%)	< 0.001
Average diameter [mm]	27.8 ± 4.1	25.4 ± 4	6.7% (± 3.5%)	< 0.001
Perimeter [mm]	92.9 ± 13.7	86.9 ± 14	4.8% (± 4.3%)	< 0.001
Area [mm^2^]	587 ± 169	487 ± 144	13.7% (± 6.8%)	< 0.001
Diameter based on area [mm]	27.1 ± 4.0	24.6 ± 3.7	7.2% (± 3.7%)	< 0.001
Relative area	1.1 ± 0.1	0.9 ± 0.1	13.8% (± 8.3%)	< 0.001
Asymmetry ratio	1.3 ± 0.2	1.1 ± 0.1	12.8% (± 10.8%)	< 0.001

*Values calculated based on all data available for each phase; ^†^relative difference compared to the maximum value; ^**ǂ**^values calculated based on the number of cases available per comparison of the means; values for relative area and asymmetry ratio were calculated with Wilcoxon signed-ranked test

### Variation of annulus parameters during the cardiac cycle per type of BAV

**Fig. 5:**
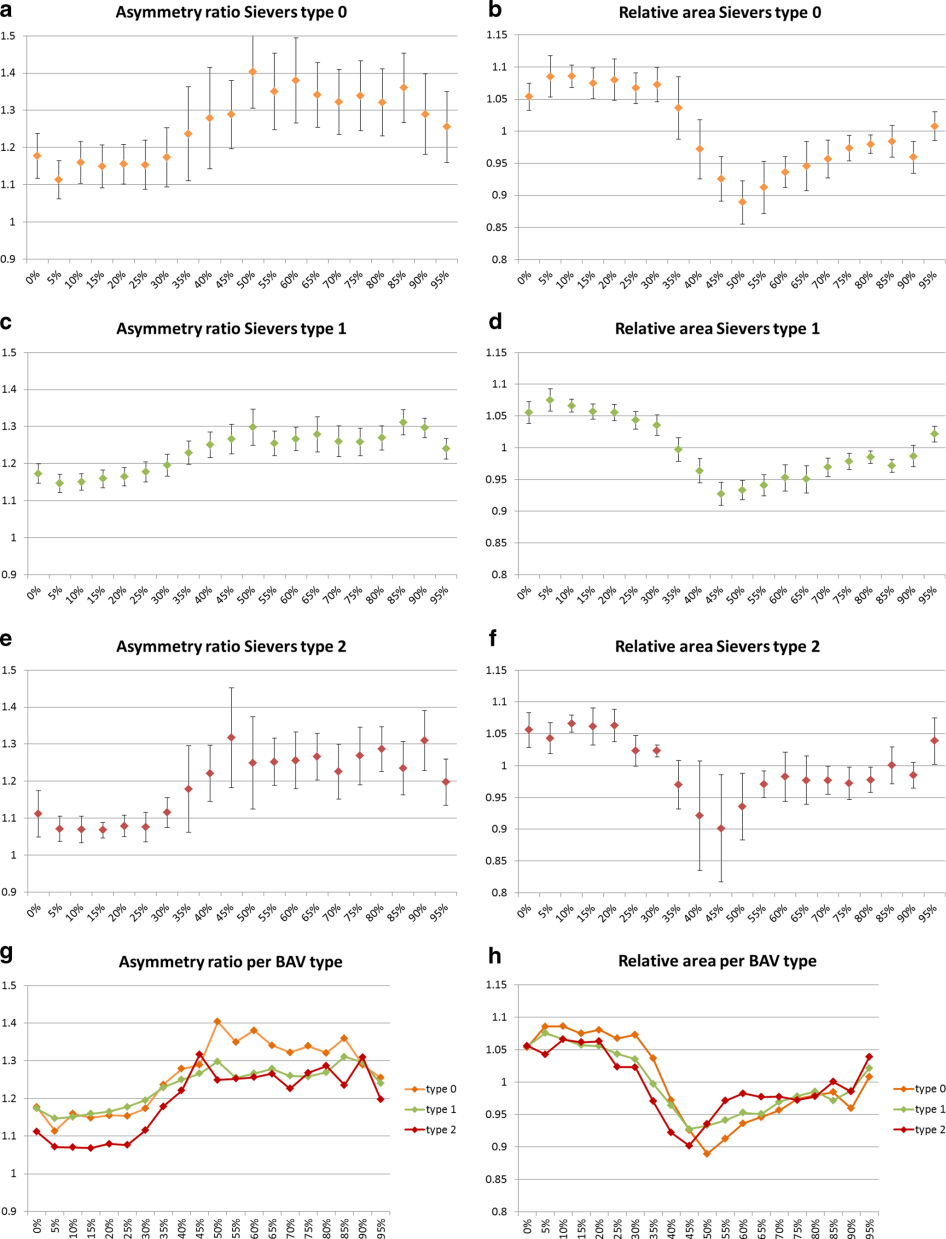
Mean AR and RA per phase and valve type. Vertical bars indicate 95% CI

When analyzed separately, all three types of BAV showed analogous dynamic changes (Table 3 and Fig. 5). All assessed parameters showed significant differences over time, with the exceptions of the maximum diameter for type 2 valves (*p* = 0.15) and maximum diameter and perimeter for type 0 valves (*p* = 0.2 and *p* = 0.2 respectively) that did not show significant differences.

**Table 3:** Changes of the annulus parameter values during the cardiac cycle per valve type

	Max value*	Min value*	Relative difference [%]†ǂ (± SD of the difference)	*p* valueǂ
				Min vs Max
Maximum diameter [mm]				
Sievers type 0	30.4 ± 3.8	28.6 ± 4.2	2.3% (± 4.7%)	0.207
Sievers type 1	30 ± 4.6	27.9 ± 4.1	3.9% (± 5.2%)	0.001
Sievers type 2	32.3 ± 2.9	29.8 ± 3.7	2.7% (± 3.4%)	0.15
Minimum diameter [mm]				
Sievers type 0	25.8 ± 4.1	20.6 ± 3.9	19.5% (± 4.5%)	< 0.001
Sievers type 1	25.5 ± 3.8	22.2 ± 3.8	11.6% (± 7.4%)	< 0.001
Sievers type 2	29.6 ± 2.2	23.5 ± 3.3	19.4% (± 9.6%)	0.028
Average diameter [mm]				
Sievers type 0	27.8 ± 4.2	24.6 ± 3.7	7.3% (± 4.7%)	0.004
Sievers type 1	27.5 ± 4.1	25 ± 3.8	7% (± 3.4%)	< 0.001
Sievers type 2	30.6 ± 2.2	27.1 ± 2.8	7.6% (± 4.8%)	0.028
Perimeter [mm]				
Sievers type 0	93.4 ± 14.3	83.7 ± 11.8	6% (± 6%)	0.027
Sievers type 1	91.8 ± 14.7	85.8 ± 13.5	5% (± 4.9%)	< 0.001
Sievers type 2	102.5 ± 8.1	93.7 ± 11.9	3.5% (± 5.9%)	0.229
Area [mm^2^]				
Sievers type 0	584 ± 168	439 ± 123	17.9% (± 6.5%)	0.001
Sievers type 1	574 ± 173	475 ± 141	13.2% (± 6.6%)	< 0.001
Sievers type 2	691 ± 117	543 ± 123	14.2% (± 10.1%)	0.042
Diameter based on area [mm]				
Sievers type 0	27 ± 3.9	23.4 ± 3.3	9.5% (± 3.5%)	< 0.001
Sievers type 1	26.7 ± 4.0	24.3 ± 3.7	6.9% (± 3.6%)	< 0.001
Sievers type 2	29.6 ± 2.6	26.1 ± 3.0	7.5% (± 5.3%)	0.038
Relative area				
Sievers type 0	1.1 ± 0.1	0.9 ± 0.0	16.4% (± 6.0%)	< 0.001
Sievers type 1	1.1 ± 0.0	0.9 ± 0.1	13.4% (± 6.5%)	< 0.001
Sievers type 2	1.1 ± 0.1	0.9 ± 0.1	17.2% (± 7.5%)	0.004
Asymmetry ratio				
Sievers type 0	1.4 ± 0.2	1.1 ± 0.1	19% (± 8.6%)	0.012
Sievers type 1	1.3 ± 0.1	1.2 ± 0.1	12.1% (± 8.8%)	< 0.001
Sievers type 2	1.3 ± 0.2	1.1 ± 0.0	17.6% (± 11.7%)	0.043

*Values calculated based on all data available for each phase; ^†^relative difference compared to the maximum value; ^**ǂ**^values calculated based on the number of cases available per comparison of the means; values for relative area and asymmetry ratio were calculated with Wilcoxon signed-ranked test

The AR was significantly different between BAV types 1 and 2 in early systole with valves type 1 showing a more elliptic annulus (*p* = 0.024 at 10%; *p* = 0.014 at 15%; *p* = 0.023 at 20%; *p* = 0.016 at 25%). Patients with valves type 0 demonstrated a significantly more elliptic annulus than type 2 at phase 10% (*p* = 0.047). The other parameters did not show significant differences between valve types at any of the time points.

### Influence of heart rate on annulus dynamics

**Fig. 6:**
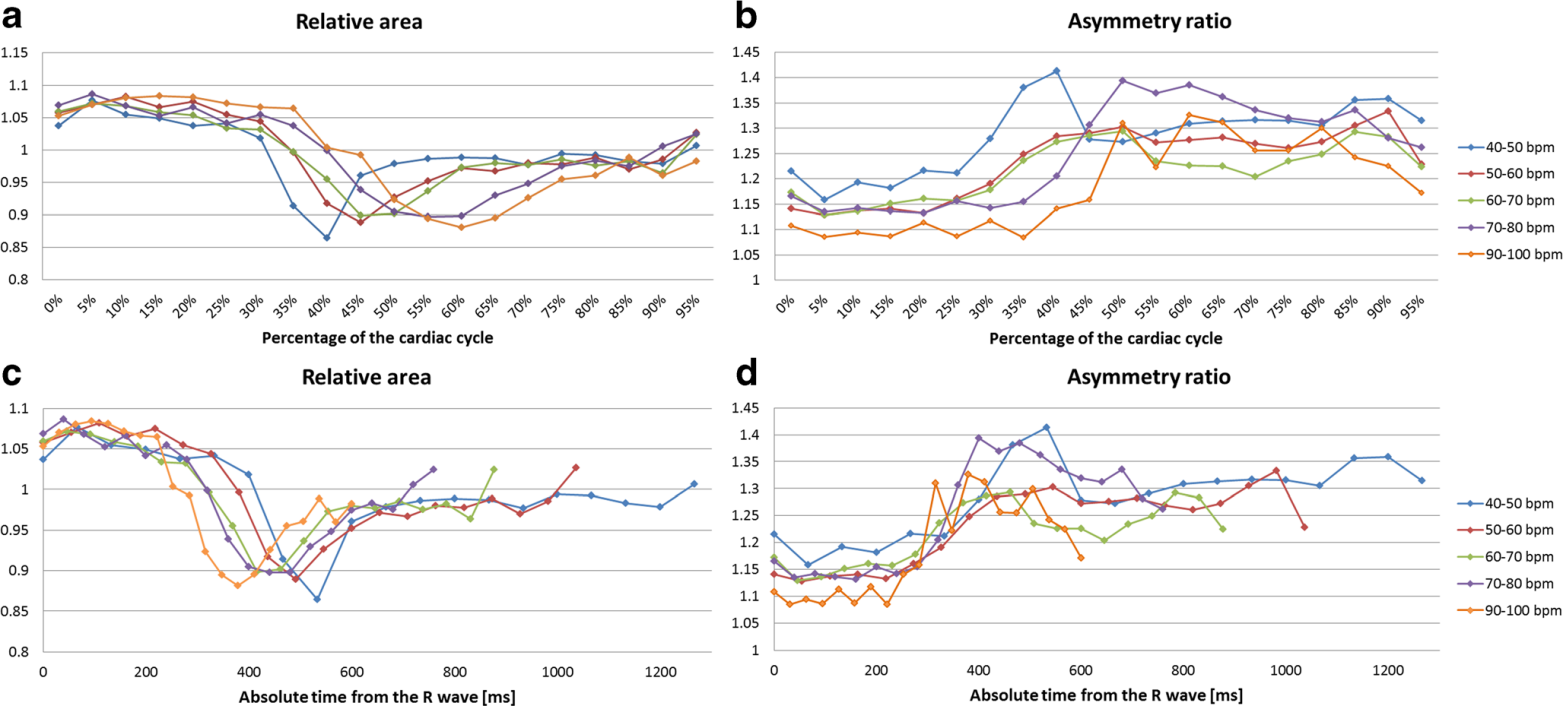
Asymmetry ratio and relative area in groups with different heart rates during successive phases of the cardiac cycle expressed in terms of percentage (**a** and **b**) and absolute time (**c** and **d**)

Scans were divided in 7 different categories based on heart rate (Table 4). All groups showed a smaller area between 40 and 60% of the cardiac cycle (Fig. 6); however, the smallest values were reached earlier in the group with the slowest heart rate.

**Table 4:** Classification of scans based on heart rate

Patients’ heart rate at the level of the aortic valve	*n*
- 40–50	6
- 50–60	15
- 60–70	16
- 70–80	10
- 80–90	4
- 90–100	4
- 100–110	2

### Intra- and inter-observer variability

Intra- and inter-observer variabilities were good with all mean differences of ≤ 0.5 ± 0.6 mm and ≤ 1 ± 1.2 mm for maximum and minimum diameter and ≤ 8.1 ± 25.9 mm^2^ and ≤ 18.2 ± 17.9 mm^2^ for the area respectively.

## Discussion

In this study, we described a standardized way to determine the annulus plane in patients with a BAV and demonstrated that the BAV annulus undergoes significant changes in shape and size during the cardiac cycle. Overall, the annulus showed a larger area with an almost circular shape in systole and a smaller area with an elliptic shape in diastole. Each of the BAV types showed analogous morphological changes. However, type 1 valves had a more elliptic annulus in early systole.

The term BAV includes a spectrum of morphological alterations of the aortic valve that have in common the resulting presence of only two functional cusps. BAV are commonly divided based on the classification system introduced by Sievers et al depending on the presence and number of raphe [1]. A raphe represents the line of fusion between two cusps that are often unequal in size. Different classification systems have been proposed highlighting the variability in morphology and difficulty of categorization of BAV patients [25]. Furthermore, the aortic root is asymmetrically and characteristically enlarged in 58% of cases [26].

In the last years, a greater interest in the annulus of BAV patients has emerged, reflecting the increasing resort to TAVI as a therapeutic option in this population. Indeed, the most important entity to be assessed is the ring where the deployed valve will be anchored. This ring, commonly referred to as “annulus,” contours the level with the smallest area along the junction between the LVOT and the aortic root but does not correspond to a well-defined anatomical structure [20, 27]. Recent guidelines (intended for patients with TAV) indicate that the annulus should be identified on MPR as the plane passing through the three hinge points of the cusps [18]. Little is known how the modified anatomy of BAV patients affects the definition of the annulus plane [21, 22]. As a consequence, the abovementioned technique based on the three hinge points has been previously applied also to datasets of patients with BAV regardless of valve type [13, 28–30]. We did not find this method feasible for all BAV patients. First, in patients with BAV valve type 0, there are only two cusps, and therefore, in most cases, only two hinge points (or in few cases two of the three are very close). Since there are infinite planes that pass through two points, more criteria to identify the correct annulus plane had to be introduced as detailed above in the Methods. Contrary to other previously published works, we proposed a combination of multiple criteria for manual identification of the annulus, taking into account the difficulty to visually identify the smallest area as well as the variability of aortic and LVOT characteristics during the cardiac cycle. Furthermore, in patients with BAV type 1 (and possibly type 2), one of three cusps is often malformed and/or underdeveloped resulting in a distortion of the normal anatomy of the aortic root. Therefore, the plane passing through the three hinge points might not be representative of the desired plane where to deploy the valve. To the best of our knowledge, we have been the first to acknowledge this intrinsic pitfall for the identification of the annulus plane in BAV type 1 and to propose a feasible solution. Interestingly, this methodology can be directly applied to any cardiac phase and notwithstanding the classification system employed for BAV, since it is based on visual assessment of the number of hinge points and cusps symmetry. With mean differences of ≤ 1 ± 1.2 mm for the maximum diameter, inter- and intra-observer variability of the measurements were very good suggesting a good reproducibility of the technique.

Whether reported underexpansion of TAVI prosthesis in BAV patients [28] and increased frequency of paravalvular leakage [12, 31] could be due to an overestimation of the annulus size related to the application of the standard methodology for its identification is yet to be confirmed. Also, the potential benefit of our methodology on clinical outcomes has yet to be proven. However, the pressing need to address the issue of correct and specific identification of the annulus in BAV patients seem apparent and of the utmost importance. The application of a specifically tailored methodology such as the one we propose, both in clinical and research settings, will likely improve the standardization of annulus measurements in BAV patients, and thereafter, provide accurate and appropriate sizing data as basis for future guidelines.

In patients with TAV, the annulus has an elliptic shape that undergoes conformational changes during the cardiac cycle as well as significant variation in sizing parameters [7]. In fact, although with some controversy attributable to the better quality and reproducibility of diastolic images [19], literature and guidelines suggest that measurements should be performed on images obtained in systole due to larger annular sizes in this phase [18, 32]. Our study demonstrated the occurrence of significant changes in sizing parameters during the cardiac cycle in all types of BAV. The RA, minimum diameter, and area showed the most pronounced differences (13.8% and 13.7%), with all highest values in the phases between 0 and 10%, while the maximum diameter demonstrated the smallest difference (3.6%). The relative difference of 7.2% found for the area-derived diameter corresponded to an absolute difference of 1.9 mm between systole and diastole. Considering that each prosthetic valve size can be fitted in annuli presenting a range of diameters of 3 mm at maximum (or 2 mm depending on the type of valve employed), a 2 mm difference could imply a different choice of valve size.

Among the reasons that led to consider BAV patients at high risk for TAVI procedures are some characteristics of the aortic annulus such as a more elliptic shape than in patients with TAV. This different conformation has been suggested as the cause for the higher rate of paravalvular regurgitation in BAV patients [10, 14, 33, 34]. With an AR > 1 at all phases and for all BAV types, but significantly more in early systolic phase for valves type 1, our study demonstrated that the annulus of BAV patients has an elliptic shape. However, the overall and per valve type maximum average AR in our study (maximum AR: 1.3 ± 0.2 overall; 1.4 ± 0.2 for valves type 0; 1.3 ± 0.1 for valves type 1; 1.3 ± 0.2 for valves type 2) are comparable to those found in patients with TAV[6, 8, 27]. Furthermore, compared to already published data, the results of our study confirm that the annulus has larger dimensions in BAV patients (maximum diameter in systole: 30.22 ± 4.48 mm overall; 30.42 ± 3.83 mm for valves type 0; 30.04 ± 4.61 mm for valves type 1; 32.34 ± 2.93 mm for valves type 2) than in patients with TAV [7, 28–30]. Data on TAV show that annulus dynamic characteristics are similar in normal and stenotic valves [6]. Although data from the present study are not sufficient to draw a conclusion in this sense, we expect the same to be true for BAV patients. Considering that these patients often have a dilation of the aorta, we could even expect them to develop a larger annulus later in life.

Hemodynamic characteristics might influence the mechanics of the annulus. Therefore, we investigated the influence of the patients’ heart rate at the moment of the CT examination. CT images were reconstructed based on the percentage of the cardiac cycle and therefore the absolute time span between phases depended on the heart rate of the patient at the moment of the acquisition. Our results suggest that, in patients with a higher heart rate, changes in area of the aortic annulus occur at a shorter absolute time, which, however, corresponds to a later phase of the cardiac cycle. Therefore, to ensure that the largest sizing parameters are detected for all heart rates, measurements should be performed in early systolic phase and within 200 ms from the R wave.

The biggest limitation of the present study is the impossibility to compare planes and measurements obtained with the new method to a reference standard. However, for the annulus, even in TAV patients, there is no reference standard. In fact, measurements performed in operating rooms are subject to limitations including non-physiological conditions and the use of an imprecise measurement tool and, furthermore, values of implanted valve size are influenced by other additional factors. It could be advocated that measurements realized according to the new proposed method should have been compared to those obtained with the standard method. This would be impossible for BAV type 0 for the abovementioned reasons. Moreover, although theoretically possible for asymmetric valves type 1, it would not yield any significant meaning as the method based on the identification of the three hinge points results in the definition of a plane that does not seem to correspond to the deployment area of the valve. Furthermore, the new method was applied in only 9 cases of type 1 BAV. Therefore, our method has to be tested otherwise in a larger cohort of patients. Second, the mean age of the study population was relatively low with a limited number of severely stenotic aortic valves. Obviously, this limits the direct applicability of our findings to patients evaluated for TAVI. However, in TAV patients, similar changes in annulus shape and dimensions were demonstrated for both healthy and stenotic valves [6]. Moreover, due to the relatively limited number of patients per type of BAV valve and, to some extent, to the excluded phases, ANOVA models for repeated measurements were not applicable and comparison between valve types had to be performed per time points. Another limitation that has to be considered is the thickness of the reconstructions (1.5 mm) which might have influenced the precision of the measurements, although, in our opinion, not so much as to change the results of the study.

To conclude, as assessed based on the proposed refined method for the identification of BAV annulus, in all three BAV types, the annulus has an elliptical shape that undergoes significant size and conformational changes with bigger dimensions in systole and a more elliptical appearance in the diastolic phase. The only exception is type 1 BAV that showed a more elliptical shape in systole as compared to type 2 valves. Our study sheds light on previously unexplored physiology of a multifaceted congenital disease. Furthermore, once the proposed method is validated against valve implantation outcomes and in patients with more severe valve dysfunction in further studies, our findings could guide and improve pre-interventional measurements.

## Supplementary Information

ESM 1 (DOCX 27 kb)

## Abbreviations

AR: Asymmetry ratio
BAV: Bicuspid aortic valve
LCC: Left coronary cusp
LVOT: Left ventricular outflow tract
NCC: Non-coronary cusp
RA: Relative area
RCC: Right coronary cusp
TAV: Tricuspid aortic valve
TAVI: Transcatheter aortic valve implantation
VR: Volume rendering

## Declarations

### Guarantor

The scientific guarantor of this publication is Ricardo PJ Budde.

### Statistics and biometry

Sara Baart kindly provided statistical advice for this manuscript.

### Study subjects or cohorts overlap

Some study subjects or cohorts have been previously reported in van den Hoven AT, Mc-Ghie JS, Chelu RG, Duijnhouwer AL, Baggen VJM, Coenen A, et al Transthoracic 3D echocardiographic left heart chamber quantification in patients with bicuspid aortic valve disease. Int J Cardiovasc Imaging. 2017;33(12):1895-9030) Lidia R. Bons, Anthonie L. Duijnhouwer, Sara Boccalini, Allard T. Van Den Hoven, Maureen J. Van Der Vlugt, Raluca G. Chelu, Jackie S. McGhie, Isabella Kardys, Annemien E. Van Den Bosch, Hans-Marc J. Siebelink, Koen Nieman, Alexander Hirsch, Craig S. Broberg, Ricardo P.J. Budde, Jolien W. Roos-Hesselink , Intermodality variation of aortic dimensions: How, where and when to measure the ascending aorta. Ijca (2018), 10.1016/j.ijcard.2018.08.067. However, there is no overlap in objectives or reported data since the aortic annulus was not analyzed in any of these papers.

### Methodology

• prospective

• observational

• performed at one institution
