# Supplementary material for: Bicuspid aortic valve annulus: assessment of geometry and size changes during the cardiac cycle as measured with a standardized method to define the annular plane
Source: Eur Radiol. 2021 Apr 24;31(11):8116–29. doi: 10.1007/s00330-021-07916-8 (PMC8523432; doi:10.1007/s00330-021-07916-8)
Supplement: Supplementary file 1 — (DOCX 27 kb) [file 330_2021_7916_MOESM1_ESM.docx]

**Supplemental material-** CT scans characteristics

| **CT protocol –scan parameters** |  |
| --- | --- |
| Scan type | Retrospective spiral |
| Collimation | 192*0,6mm |
| Rotation time | 0,250 sec. |
| Pitch | adjusted to heart rate |
| Feed/rot | adjusted to heart rate |
| Reference mAs/rot | 150 |
| mA modulation | Yes, CARE Dose 4D |
| Reverence kVp | 120 |
| kVp modulation | Yes, CARE kV |
| ECG pulsing | 1-40% |
| **CT protocol – contrast** |  |
| Contrast | Yes |
| Type | Iodixanol* 320mgI/ml |
| Bolus | 65ml at 5ml/sec. |
| Chaser (30% mix ratio) | 40ml at 5ml/sec. |
| Delay | Bolus-tracking |
| Kernel | Bv36 |
| ADMIRE strength | 3 |
| **CT protocol - reconstruction** |  |
| Function Recon1 slice width/increment | 1,5 mm/1,0mm |
| Function Recon1 FOV & matrix | 190mm 256 matrix |
| Function Recon1 ECG phase % | 0-100% at 5% interval |
